# Discriminative Ability of TyG, TyG-WC, BAI, FGIR, and QUICKI Indexes in Identifying Metabolic Syndrome in a Pediatric Population with Obesity

**DOI:** 10.3390/metabo16060415

**Published:** 2026-06-14

**Authors:** Sofia Tamini, Adele Bondesan, Diana Caroli, Francesca Frigerio, Alessandro Sartorio

**Affiliations:** Experimental Laboratory for Auxo-Endocrinological Research, Istituto Auxologico Italiano, IRCCS, Piancavallo, 28824 Verbania, Italy; a.bondesan@auxologico.it (A.B.); d.caroli@auxologico.it (D.C.); f.frigerio@auxologico.it (F.F.); sartorio@auxologico.it (A.S.)

**Keywords:** obesity, pediatric obesity, children, adolescents, metabolic syndrome, MetS, TyG, TyG-WC, FGIR, QUICKI

## Abstract

**Background/Objectives**: Pediatric obesity is closely associated with metabolic syndrome (MetS), a condition linked to increased cardiometabolic risk. Early identification of high-risk individuals remains challenging. This study aimed to evaluate the diagnostic performance of selected anthropometric, metabolic dysfunction and insulin resistance indexes for identifying MetS in children and adolescents with obesity. **Methods**: In this retrospective, cross-sectional, single-center study, 758 children and adolescents with obesity (mean age 14.8 ± 2.1 years; 59.9% females) hospitalized for a body weight-reduction program were included. MetS was defined according to International Diabetes Federation criteria, in which central obesity is a mandatory diagnostic component. The triglyceride–glucose (TyG), TyG–waist circumference (TyG-WC), body adiposity index (BAI), fasting glucose-to-insulin ratio (FGIR), and quantitative insulin sensitivity check index (QUICKI) were calculated. Receiver operating characteristic curve analysis was used to assess their discriminative ability. **Results**: The prevalence of MetS was 27.8% and was significantly higher in males than females (34.9% vs. 23.1%, *p* < 0.0001). TyG and TyG-WC showed the best discriminative performance (AUC 0.75 and 0.76, respectively), although with only moderate sensitivity and specificity. FGIR and QUICKI demonstrated lower accuracy (AUC 0.64 and 0.63), whereas BAI showed no discriminative ability (AUC 0.48). These findings were consistent across sexes, although sex-specific differences in both MetS prevalence and optimal cut-off values were observed. Correlation analyses confirmed moderate associations between TyG-based indexes and MetS, whereas other indexes showed weaker relationships. **Conclusions**: In the present cohort of children and adolescents with obesity, TyG and TyG-WC showed the best performance in identifying MetS compared with the other evaluated indexes. However, their performance remained moderate, and the proposed cut-off values require validation in independent populations. These indexes may represent simple supportive screening and risk-stratification tools but should be used alongside comprehensive clinical assessment and established diagnostic criteria rather than as stand-alone diagnostic measures.

## 1. Introduction

Childhood obesity is recognized as a leading public health challenge of the 21st century. The World Health Organization European Childhood Obesity Surveillance Initiative recently reported data from its sixth round (2022–2024), including approximately 470,000 children across 37 countries [1]. Although many children with overweight or moderate obesity may remain asymptomatic during early life, children with severe obesity frequently present early clinical complications, including an unfavorable cardiometabolic profile, early vascular alterations, and evidence of subclinical atherosclerosis [2]. Obesity is a major determinant of insulin resistance (IR) and plays a central role in the development of metabolic syndrome (MetS), a condition characterized by the coexistence of multiple metabolic abnormalities [3,4]. Additionally, MetS is closely linked to an elevated risk of cardiovascular disease and type 2 diabetes (T2D) [5]. Furthermore, large-scale prospective studies have shown that overweight status frequently persists from childhood into adulthood, with children and adolescents with excess weight having an approximately fivefold increased risk of adult obesity and nearly 80% remaining obese later in life [3,6].

Consequently, the rising burden of pediatric obesity has been paralleled by an increase in the prevalence of MetS. Recent studies indicate that the prevalence of MetS among obese children has reached concerning levels, with highly variable estimates (approximately 3–66%) depending on diagnostic criteria and population characteristics. Nonetheless, most children and adolescents with obesity, approximately 90%, exhibit at least one component of MetS [7,8].

The strong association between pediatric obesity and MetS highlights the need for early detection and timely intervention to mitigate long-term complications [7]. However, defining MetS in children and adolescents remains controversial, mainly due to the absence of universally accepted pediatric cut-offs for its individual components [5,9,10].

Taken together, these limitations underscore the need for practical and reliable tools to improve early identification of metabolic risk in pediatric populations [7]. In this context, non-invasive surrogate indexes represent a promising approach for large-scale screening and early risk stratification.

Over the years, various indexes have been developed for this purpose, the first being the body mass index (BMI), which is widely used in clinical practice [11]. However, single-dimensional metrics such as BMI do not fully capture the complexity of obesity. In particular, BMI cannot distinguish between fat and lean mass nor assess fat distribution, key determinants of cardiometabolic risk [12,13,14]. Therefore, although BMI remains a useful tool for initial screening, it does not adequately reflect inter-individual variability in metabolic risk [15].

To overcome these limitations, several alternative indexes that combine anthropometric and biochemical parameters have been proposed. Given the central role of insulin resistance (IR) in the pathophysiology of MetS, indexes reflecting IR may serve as valuable tools for early risk identification. Among these, the most extensively investigated include the triglyceride–glucose index (TyG), and in particular its modified form incorporating waist circumference (TyG-WC), as well as the Body Adiposity Index (BAI), the fasting glucose-to-insulin ratio (FGIR), and the Quantitative Insulin Sensitivity Check Index (QUICKI).

The TyG index is increasingly recognized as a surrogate marker for IR and metabolic dysfunction, with demonstrated predictive value for IR and MetS in both adults and pediatric populations. Several pediatric studies have reported significant associations between TyG-derived indexes and metabolic abnormalities, including insulin resistance, MetS, and metabolic dysfunction-associated liver disease. However, the magnitude of these associations and the proposed diagnostic cut-off values have varied considerably across studies, likely reflecting differences in age, pubertal development, obesity severity, ethnicity, and the diagnostic criteria adopted for MetS and insulin resistance [16,17,18,19]. In an attempt to improve risk discrimination, modified TyG indexes, which incorporate anthropometric measures such as waist circumference (TyG-WC), have been proposed and, in some pediatric cohorts, have shown better diagnostic performance than TyG alone [19,20,21]. These indexes integrate lipid and glucose parameters with anthropometric measures, providing a practical approach for risk identification. Nevertheless, comparative evidence remains limited, and it is still unclear whether the addition of anthropometric measures consistently improves performance across different pediatric populations.

The BAI has been proposed as an alternative anthropometric measure of adiposity, derived from hip circumference and height, and can estimate body fat percentage in adults [22]. Previous studies have reported associations between BAI and MetS in adults [23,24,25,26]. However, evidence in pediatric populations is considerably more limited and conflicting. Because BAI primarily reflects body adiposity rather than metabolic dysfunction, its ability to identify children with an adverse metabolic profile remains uncertain. Although this poses a potential limitation, BAI is a simple anthropometric indicator of body fatness [27,28] and therefore provides an opportunity to compare the performance of adiposity-based measures against indexes reflecting insulin sensitivity and metabolic dysfunction.

The FGIR and QUICKI indexes represent alternative approaches based on fasting glucose and insulin measurements and have been widely used as surrogate markers of insulin sensitivity. The FGIR index has been used as a marker of insulin sensitivity, including in pediatric settings, and has shown potential associations with MetS [29,30,31]. The QUICKI index, also derived from fasting glucose and insulin levels, was initially developed to identify insulin resistance and has been suggested as a predictor of MetS [30,32,33,34]. Although FGIR and QUICKI are established surrogate markers of insulin sensitivity [35,36], their clinical utility in pediatric obesity remains uncertain for MetS discrimination. Likewise, evidence regarding the usefulness of BAI in pediatric populations is limited and less consistent than in adults.

Despite growing interest in these surrogate markers, most available evidence has focused on individual indexes or specific outcomes, such as insulin resistance, hepatic steatosis, or isolated cardiometabolic abnormalities, and direct comparative analyses across multiple indexes in pediatric populations remain limited. In particular, data on the relative performance of indexes reflecting different aspects of metabolic dysfunction, such as lipid-glucose integration (TyG-based indexes), anthropometry (BAI), and insulin sensitivity (FGIR and QUICKI), are still scarce and inconsistent. Notably, our previous work primarily focused on TyG-derived indexes in relation to primary hepatic and secondary metabolic outcomes, highlighting their potential clinical utility [19]. However, while those findings supported the clinical utility of TyG-based markers, that approach did not allow a comprehensive comparison with other widely used indexes of insulin sensitivity and adiposity within the same population. The present study was specifically designed to address this gap by providing a broader, direct comparison and more integrative evaluation of multiple indexes within a large cohort of children and adolescents with obesity.

Therefore, evaluating the diagnostic performance of these indexes in children and adolescents is essential for establishing their clinical utility as non-invasive screening tools.

For these reasons, this study aimed to evaluate the predictive performance of the TyG, TyG-WC, BAI, FGIR and QUICKI indexes for identifying MetS in a large cohort of children and adolescents with obesity.

## 2. Materials and Methods

### 2.1. Study Population

The present retrospective, cross-sectional, single-center study was conducted among children and adolescents with obesity admitted to a three-week multidisciplinary body weight reduction program (BWRP) at the Division of Auxology, Istituto Auxologico Italiano, IRCCS, Piancavallo-Verbania, between March 2018 and September 2022. The study dataset was retrospectively extracted from the institutional clinical database and included routinely collected clinical, anthropometric, and biochemical data obtained during hospital admission as part of the standard diagnostic evaluation performed within the BWRP.

Eligibility criteria included: (i) age between 10 and 18 years; (ii) both sexes; and (iii) a body mass index (BMI) standard deviation score (SDS) > 2, according to Italian reference growth charts [37]. Participants with syndromic or monogenic obesity (e.g., Prader–Willi syndrome), endocrine disorders known to affect body weight (e.g., hypothyroidism, Cushing syndrome, growth hormone deficiency), or obesity secondary to chronic pharmacological treatments (e.g., glucocorticoids, antipsychotics, or antiepileptic drugs) were excluded based on medical history, clinical evaluation, and available medical records. Additionally, participants were also excluded in case of incomplete clinical or biochemical data required for the calculation of the investigated indexes.

All subjects underwent a standardized clinical evaluation, including medical history collection, physical examination, routine hematological and biochemical assessments, and urine analysis.

The study protocol was approved by the Ethical Committee no. 5, Lombardy Region, Italy, Milan, Italy (ethical code number: 17/25, date of approval: 25 March 2025; acronym: INDEMETSPED). At hospital admission, written informed consent for the anonymous use of clinical, anthropometric, and biochemical data for research purposes was obtained from all participants and their parents or legal guardians.

### 2.2. Anthropometry and Puberal Stage

At admission, height and body weight (BW) were measured according to internationally standardized procedures [38] using a calibrated stadiometer scale (Wunder Sa.Bi., WU150, Trezzo sull’Adda, Italy), with participants wearing light clothing and no shoes. BMI was subsequently calculated as weight (kg)/height (m)^2^.

Waist circumference (WC) and hip circumference (HC) were measured using a non-elastic flexible measuring tape. WC was measured at the midpoint between the lower costal margin and the iliac crest, whereas HC was recorded at the level of maximum gluteal protuberance.

Pubertal development was assessed at admission using Tanner staging, evaluated by trained pediatric endocrinologists during physical examination [39,40].

### 2.3. Laboratory and Clinical Parameters

Venous blood samples (approximately 10 mL) were obtained in the early morning after an overnight fast and collected into standard serum tubes. Fasting plasma glucose (FPG), total cholesterol (T-C), high-density lipoprotein cholesterol (HDL-C), and triglycerides (TG) were analyzed in the same certified laboratory using standardized methods. Fasting plasma insulin (FPI) concentration was measured using a chemiluminescent immunometric assay (Elecsys Insulin, Roche Diagnostics, Monza, Italy), with a sensitivity of 0.2 μIU/mL.

Systolic (SBP) and diastolic (DBP) blood pressure were assessed on the dominant arm using an aneroid sphygmomanometer (TemaCertus, Milan, Italy) with appropriately sized cuffs. Two measurements were obtained at 3 min intervals. The average of the two readings was calculated and rounded to the nearest 5 mmHg.

Metabolic syndrome (MetS) was defined according to the International Diabetes Federation (IDF) criteria for children and adolescents [41]. Specifically, MetS was diagnosed in the presence of central obesity, defined as WC ≥ 90th percentile for subjects aged <16 years, and ≥94 cm in males and ≥80 cm in females aged ≥16 years, together with at least two of the following criteria: (i) TG ≥ 150 mg/dL or in ongoing treatment for dyslipidemia; (ii) HDL-C < 40 mg/dL in subjects aged <16 years, <40 mg/dL in males ≥16 years, or <50 mg/dL in females ≥16 years, or in ongoing treatment for dyslipidemia; (iii) SBP ≥ 130 mmHg or DBP ≥ 85 mmHg; (iv) FPG ≥ 100 mg/dL or diagnosis of T2D.

### 2.4. Indexes

The five indexes were calculated according to the following formulas:

- TYG (males/females) [16,42] = ln [(TG(mg/dL) * FPG(mg/dL))/2]
- TYG-WC (males/females) [43] = TYG * WC
- BAI (males/females) [22] = (HC(cm)/height(m)^1,5^)-18
- FGIR (males/females) [29,44] = FPG(mg/dL)/FPI(μU/mL)
- QUICKI (males/females) [32] = 1/[log(FPI(μU/mL)) + log (FPG(mg/dL))]

### 2.5. Statistical Analysis

Continuous variables are reported as mean ± standard deviation, while categorical variables as absolute and relative frequencies. Normality was verified using the Shapiro–Wilk test.

Receiver operating characteristic (ROC) curve analysis was performed to evaluate each index’s ability to discriminate the presence of MetS, and the corresponding area under the curve (AUC) was calculated. Optimal cut-off values for each index were determined using the Youden index [45]. All analyses were conducted both in the overall population and after stratification by sex.

Participants were categorized according to the presence (MetS+) or absence (MetS−) of MetS, and sex (females and males). Between-group comparisons (MetS+ vs. MetS− and females vs. males) were performed using the unpaired *t*-student test for continuous variables or Fisher’s exact test for categorical variables.

Pearson’s correlation analysis was used to explore the relationships between each index and the anthropometric and metabolic parameters considered. Correlation strength was interpreted as follows: r value < ±0.10: negligible correlation; ±0.11–±0.39: weak correlation; ±0.40–±0.69: moderate correlation; ±0.70–±0.89: strong correlation and values > ±0.90: a very strong correlation [46].

A *p* < 0.05 was considered statistically significant.

## 3. Results

A total of 760 potentially eligible participants were identified. Two subjects were excluded because of incomplete clinical or biochemical data required for the calculation of the investigated indexes, resulting in a final study population of 758 children and adolescents with obesity. The study participant flow chart is illustrated in Supplementary Figure S1.

Females represented 59.9% of the sample (n = 454), while males accounted for 40.1% (n = 304). The mean age of the total cohort was 14.8 ± 2.1 years, while the mean BMI was 37.9 ± 6.2. The prevalence of MetS was 27.8% (211/758). Participants were stratified by the presence/absence of MetS (MetS+ and MetS−, respectively).

Table 1 shows the main characteristics of the entire study group and of the two subgroups (MetS+ and MetS−).

As expected, most cardiometabolic parameters were significantly worse in the MetS+ subgroup. Subjects in the MetS+ subgroup showed significantly higher values of anthropometric measures (WC, HC, BW, height, BMI), blood pressure (SBP and DBP), fasting plasma insulin, TG, and TyG-derived indexes (all *p* < 0.0001) compared with subjects without MetS. Moreover, HDL-C, FGIR, and QUICKI were significantly lower in the MetS+ subgroup (all *p* < 0.0001). Lastly, Tanner stage was higher in the MetS+ subgroup (*p* < 0.0001). No significant differences were observed for fasting glucose, T-C, and BAI between groups.

The whole study population was also divided into males and females. Table 2 shows the characteristics of the male and female subgroups.

The prevalence of MetS was significantly lower in females (23.1%) than in males (34.9%; *p* < 0.0001).

Mean age did not differ significantly between sexes (14.8 ± 2.1 years in females vs. 14.6 ± 2.2 years in males; *p* = ns). No significant sex differences were observed for HC, BMI, fasting plasma glucose and insulin, T-C, FGIR and QUICKI. By contrast, males exhibited significantly higher WC (120.1 ± 15.1 cm vs. 112.0 ± 13.5 cm; *p* < 0.0001), BW (108.1 ± 26.4 kg vs. 97.2 ± 18.7 kg; *p* < 0.0001), height (167.0 ± 11.4 cm vs. 160.3 ± 7.4 cm; *p* < 0.0001), SBP (128.4 ± 12.7 mmHg vs. 123.5 ± 12.1 mmHg; *p* < 0.0001) and DBP (79.7 ± 8.3 mmHg vs. 77.8 ± 7.6 mmHg; *p* < 0.01), TG (101.8 ± 41.5 mg/dL vs. 93.1 ± 40.1 mg/dL; *p* < 0.01). Likewise, TyG and TyG-WC were significantly higher in males (TyG = 4.5 ± 0.2 vs. 4.4 ± 0.2, *p* < 0.001; TyG-WC = 537.8 ± 75.5 vs. 495.9 ± 68.6, *p* < 0.0001), whereas BAI was higher in females (42.3 ± 5.4 vs. 38.0 ± 5.4, *p* < 0.0001). Additionally, females had significantly higher HDL-C (44.3 ± 10.4 mg/dL vs. 40.5 ± 10.3 mg/dL; *p* < 0.0001) and Tanner stage (4.0 ± 1.3 vs. 3.5 ± 1.4; *p* < 0.0001).

**Figure 1:**
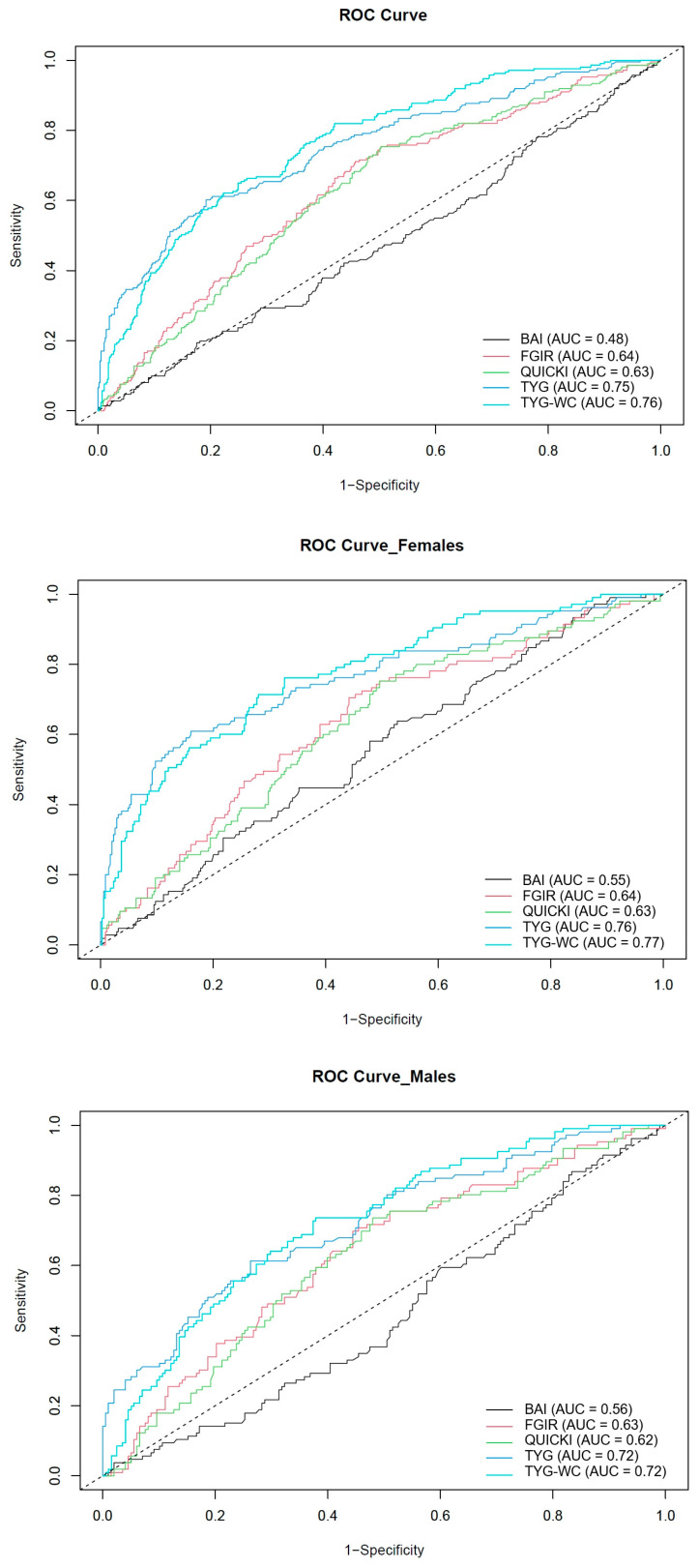
ROC curves for the indexes.

Figure 1 shows the ROC curves, while the detailed results of the ROC analysis are reported in Table 3.

ROC analyses were performed to assess the diagnostic performance of each index for identifying MetS. In the overall study population, TyG and TyG-WC showed the best discriminative ability, FGIR and QUICKI showed modest performance, and BAI showed no discriminative ability.

In detail, the TyG index showed an area under the curve (AUC) of 0.75 (95% CI: 0.71–0.79), with an optimal cut-off value of 4.54, corresponding to a sensitivity of 60.2% and a specificity of 80.2%. The TyG-WC index demonstrated a slightly higher discriminatory power, with an AUC of 0.76 (95% CI: 0.73–0.80) and an optimal cut-off of 530.07, yielding a sensitivity of 64.9% and a specificity of 75.1%. The BAI demonstrated lower discriminative power, with an AUC of 0.48 (95% CI: 0.43–0.53) and an optimal cut-off of 45.39, yielding a sensitivity of 19.4% and a specificity of 82.4%. The FGIR index showed an AUC of 0.64 (95% CI: 0.59–0.68) and an optimal cut-off of 6.2, yielding a sensitivity of 71.1% and a specificity of 54.3%. Lastly, the QUICKI showed an AUC of 0.63 (95% CI: 0.58–0.67) and an optimal cut-off of 0.34, yielding a sensitivity of 75.4% and a specificity of 49.7%.

When analyzed separately by sex, TyG and TyG-WC maintained the highest discriminative capacities in both females and males, BAI consistently showed poor performance in both sexes, while FGIR and QUICKI maintained modest discriminative ability.

In females, the TyG index exhibited an AUC of 0.76 (95% CI: 0.70–0.82) with the same cut-off of 4.54, achieving 61.0% sensitivity and 84.0% specificity. The TyG-WC index showed an AUC of 0.77 (95% CI: 0.72–0.82), with an optimal cut-off of 500.40, yielding a sensitivity of 76.2% and a specificity of 67.3%. The BAI showed an AUC of 0.55 (95% CI: 0.50–0.62) and an optimal cut-off of 40.78, yielding a sensitivity of 63.8% and a specificity of 47.3%. The FGIR index showed an AUC of 0.64 (95% CI: 0.58–0.70) and an optimal cut-off of 6.27, yielding a sensitivity of 70.5% and a specificity of 55.9%. Lastly, the QUICKI showed an AUC of 0.63 (95% CI: 0.57–0.69), and with the same optimal cut-off of 0.34, the sensitivity was 75.2%, while the specificity was 50.4%.

In males, the TyG index had an AUC of 0.72 (95% CI: 0.66–0.78) with the same cut-off of 4.54, a sensitivity of 61.3%, and a specificity of 73.7%. The TyG-WC yielded an AUC of 0.72 (95% CI: 0.66–0.78) with a cut-off of 529.39, corresponding to 73.6% sensitivity and 62.1% specificity. The BAI showed an AUC of 0.56 (95% CI: 0.50–0.62) and an optimal cut-off of 32.96, yielding a sensitivity of 86.8% and a specificity of 16.7%. The FGIR index showed an AUC of 0.63 (95% CI: 0.57–0.70) and an optimal cut-off of 5.95, yielding a sensitivity of 69.8% and a specificity of 55.6%. Lastly, the QUICKI showed an AUC of 0.62 (95% CI: 0.56–0.69), and with the same optimal cut-off of 0.33, the sensitivity was 73.6%, while the specificity was 52.0%.

Pearson correlation coefficients between each index and anthropometric/clinical variables are summarized in Supplementary Table S1.

Overall, several variables showed weak or non-significant correlations with the indexes, both in the overall population and in sex-stratified analyses.

In the whole study population, the TyG index showed a moderate correlation with MetS (r = 0.41, *p* < 0.001) and weak correlations with BW, BMI, WC, SBP and DBP. The TyG-WC index demonstrated a strong correlation with WC, BW, and BMI, as well as a moderate correlation with MetS (r = 0.41, *p* < 0.001), and weaker correlations with the remaining variables. The BAI showed a moderate correlation with BMI, HC and height, and a weak correlation with BW, WC and DBP, but was not significantly associated with MetS (r = 0.03, *p* = ns). FGIR showed weak correlations with height, BW, BMI, WC, HC, SBP, and DBP, and a weak correlation with MetS (r = 0.15, *p* < 0.001). QUICKI demonstrated a similar pattern, with weak correlations with height, BW, BMI, WC, HC, SBP and DBP and also a weak correlation with MetS (r = 0.18, *p* < 0.001).

In males, the TyG index showed a weak correlation with MetS (r = 0.37, *p* < 0.001) and weak correlations with age, height, BW, BMI, WC and SBP. The TyG-WC index demonstrated a strong correlation with WC, BW, BMI, and HC, as well as a moderate correlation with MetS (r = 0.35, *p* < 0.001), and all the other parameters. The BAI showed a moderate correlation with BMI, HC and height, a weak correlation with age, BW, WC and DBP, and no significant association with MetS (r = 0.07, ns). FGIR showed a weak correlation with all parameters, including MetS (r = 0.16, *p* < 0.001). QUICKI similarly showed weak correlations with all parameters and with MetS (r = 0.18, *p* < 0.001).

Lastly, in females, the TyG index showed a moderate correlation with MetS (r = 0.42, *p* < 0.001) and weak correlations with BW, BMI, WC and SBP. The TyG-WC index demonstrated a strong correlation with WC, BW, BMI and HC, as well as a moderate correlation with MetS (r = 0.43, *p* < 0.001), and all other parameters. The BAI showed a strong correlation with BMI and HC, a moderate correlation with BW and WC, and weak correlations with height, SBP and DBP, but was not significantly associated with MetS (r = 0.09, ns). FGIR showed a weak correlation with BW, BMI, WC, HC, SBP, DBP, and MetS (r = 0.14, *p* < 0.001). QUICKI similarly showed weak correlations with BW, BMI, WC, HC, SBP and DBP, as well as a weak correlation with MetS (r = 0.17, *p* < 0.001).

## 4. Discussion

Metabolic syndrome (MetS) is a complex condition characterized by the clustering of multiple metabolic abnormalities and is frequently observed in children and adolescents with obesity [47]. Given the increasing prevalence of pediatric obesity and MetS, and their strong association with early cardiometabolic risk, timely identification may enable early and individualized interventions aimed at preventing progression toward cardiovascular disease and T2D later in life [7,8].

For these reasons, the present study aimed to evaluate the diagnostic performance of several metabolic dysfunction and insulin resistance indexes in identifying MetS in a large cohort of children and adolescents with obesity. Overall, our findings indicate that among the evaluated indexes, TyG and TyG-WC achieved the highest discriminative ability for MetS, albeit with only moderate accuracy, whereas FGIR and QUICKI showed limited performance and BAI did not provide meaningful discrimination.

The prevalence of MetS in our cohort was 27.8%, higher in males than in females, consistent with previous reports in pediatric populations with obesity, although wide variability has been described in the literature [7,8,47]. This heterogeneity is largely attributable to differences in diagnostic criteria, age ranges, and population characteristics [7]. Notably, as observed in prior studies, a considerable proportion of individuals without a formal diagnosis of MetS still presented isolated metabolic abnormalities [7,8,47], further supporting the concept that cardiometabolic risk in pediatric obesity exists along a continuum rather than as a dichotomous condition [5,9,10].

As expected, most anthropometric and metabolic parameters were significantly worse in subjects with MetS compared to those without MetS. While the two subgroups were comparable in fasting glucose and total cholesterol, the MetS+ group showed higher BW, BMI, WC, HC, and blood pressure, as well as higher TG and fasting insulin and lower HDL-C. This pattern confirms that MetS identifies a subgroup of pediatric patients with obesity characterized by a more pronounced clustering of cardiometabolic alterations, rather than representing a distinct or isolated condition.

When anthropometric and biochemical parameters were analyzed by sex, clear differences in metabolic risk profiles emerged. Although age, BMI, fasting glucose, insulin, T-C, FGIR, and QUICKI were comparable between sexes, males exhibited higher WC, BW, height, blood pressure, and TG levels, whereas females showed higher HDL-C levels, which may offer some protective effect against metabolic dysfunction [48]. These results are consistent with accumulating evidence indicating a sexual dimorphism in cardiometabolic risk during adolescence, likely reflecting differences in body fat distribution, hormonal regulation, and metabolic adaptation during pubertal development [49].

As reported above, among the indexes evaluated, TyG and its modified form TyG-WC demonstrated the best performance in identifying MetS in our cohort. These findings are consistent with previous evidence supporting the role of TyG as a surrogate marker of metabolic dysfunction in both adult and pediatric settings [16,17,18,19]. From a pathophysiological perspective, this is not unexpected, as the TyG index integrates fasting triglycerides and glucose, two key components closely linked to insulin resistance and metabolic dysfunction [16,19,42]. The slightly better performance of TyG-WC compared to TyG alone may reflect the added value of incorporating an anthropometric parameter that reflects central adiposity, a major determinant of metabolic risk. This observation is particularly relevant in pediatric populations, where visceral fat accumulation plays a pivotal role in early metabolic impairment. However, this finding should be interpreted with caution, since waist circumference is included in both the TyG-WC formula and the IDF definition of MetS. Consequently, some degree of circularity may have contributed to the superior discriminative performance observed for TyG-WC, potentially leading to a partial overestimation of its diagnostic accuracy.

In this context, an important aspect of the present study is the direct comparison of indexes based on different conceptual approaches, namely lipid-glucose derived markers such as TyG and TyG-WC, insulin-based indexes (FGIR and QUICKI), and purely anthropometric measures (BAI), within the same pediatric population. While previous studies, including our prior work [19], have primarily focused on TyG-derived indexes in relation to specific outcomes such as hepatic outcomes, the current analysis extends this perspective by systematically evaluating their relative performance against other commonly used markers. This comparative framework is particularly relevant from a clinical perspective, as it allows a direct appraisal of which type of index, lipid-glucose–based, insulin-derived, or anthropometric, provides the most reliable discrimination of MetS within the same population and under the same conditions. This approach provides clinically meaningful information on these indexes’ relative performance that cannot be inferred when markers are analyzed separately.

In contrast, FGIR and QUICKI, despite being established markers of insulin sensitivity [29,30,31,32,33,34], showed only modest discriminative ability in our cohort. Although statistically associated with MetS, both indexes showed weak correlations and limited accuracy, suggesting that their applicability as standalone screening tools in pediatric obesity may be restricted. This finding may be explained by the complex and multifactorial nature of insulin resistance in this age group, in which dynamic physiological changes associated with growth and puberty may influence fasting insulin and glucose levels [50,51,52]. Importantly, both FGIR and QUICKI are inversely related to insulin resistance, with lower values indicating reduced insulin sensitivity. Accordingly, the significantly lower FGIR and QUICKI values observed in the MetS+ subgroup are biologically consistent with the greater degree of metabolic impairment expected in children and adolescents with MetS. These results, however, suggest that indexes relying exclusively on insulin-based measurements may be less robust in this age group than composite markers that also capture lipid metabolism. Furthermore, insulin sensitivity during childhood and adolescence is strongly influenced by pubertal maturation [53], which may contribute to the variability observed in FGIR and QUICKI values.

BAI showed no significant association with MetS and exhibited poor diagnostic performance, with an AUC close to 0.5. This result suggests that anthropometric indexes that do not incorporate metabolic parameters may fail to capture the underlying metabolic dysfunction associated with MetS. While some studies in adult populations have reported associations between BAI and cardiometabolic risk [23,24,25,26], our results do not support its use in pediatric populations with obesity, likely due to age-related differences in body composition and fat distribution. A possible explanation is that BAI was originally developed as an estimator of body adiposity rather than metabolic dysfunction and may therefore be less suitable for identifying cardiometabolic risk. Furthermore, in children and adolescents with severe obesity, marked alterations in body composition and fat distribution may reduce the ability of hip circumference-based measures to discriminate metabolic abnormalities. This may partly explain the near-random discriminative performance observed in the present cohort.

An additional relevant finding of our study is the presence of sex-related differences in metabolic risk. In particular, the prevalence of MetS was significantly higher in males than in females, although the overall performance of the indexes was broadly comparable between sexes, with some variability in cut-off values and diagnostic parameters.

These findings are consistent with growing evidence indicating a marked sexual dimorphism in cardiometabolic risk during childhood and adolescence. Although the underlying mechanisms were not directly investigated in the present study, explanations proposed in the literature include differences in body fat distribution, endocrine regulation, and metabolic responses as potential contributors. In particular, previous studies suggest that males tend to accumulate a greater proportion of visceral adipose tissue, which is more metabolically active and strongly associated with insulin resistance and systemic inflammation, whereas females generally exhibit a more favorable subcutaneous fat distribution and higher HDL-cholesterol levels. Hormonal factors may further contribute to these differences, as estrogens are known to exert protective metabolic effects by modulating lipid metabolism and insulin sensitivity, while androgens have been associated with less favorable metabolic profiles. These mechanisms may partially explain the higher prevalence of MetS observed in males and the sex-specific differences in cardiometabolic risk reported in recent pediatric studies [49,54,55]. Notably, Tanner stage was significantly higher both in participants with MetS and in females compared with males. Since pubertal development is known to influence insulin sensitivity, body composition, lipid metabolism, and cardiometabolic risk [56,57], pubertal maturation may have contributed, at least in part, to some of the observed differences between groups.

Taken together, these findings support the need for sex-specific approaches in the assessment and interpretation of metabolic risk in pediatric populations, including the potential definition of sex-specific cut-off values for metabolic indexes.

From a clinical perspective, our findings support the potential utility of TyG and TyG-WC as simple, inexpensive, and non-invasive tools for the early identification of metabolic syndrome in children and adolescents with obesity who may be at increased cardiometabolic risk. Their reliance on routinely available laboratory and anthropometric parameters makes them well suited as supportive tools for large-scale screening and early risk stratification, particularly in settings where more sophisticated methods for assessing insulin resistance are not feasible. However, given their only moderate discriminative performance, these indexes should be considered complementary to, rather than replacements for, established clinical and diagnostic criteria for MetS.

Importantly, the comparative design of this study strengthens its clinical relevance by enabling a direct comparison across commonly used indexes within the same real-world population, thus providing a clearer rationale for prioritizing specific markers in clinical screening pathways.

Despite these findings, some limitations should be acknowledged. First, the study’s retrospective, cross-sectional design does not allow causal inferences. Second, the dichotomous definition of MetS based on IDF criteria may not fully capture the continuous and complex nature of metabolic risk in pediatric populations. Third, the optimal cut-off values identified through ROC analysis were derived from the present cohort and were not internally or externally validated; therefore, their generalizability and clinical applicability require confirmation in independent populations. Furthermore, the present study was designed to compare the discriminative performance of different indexes rather than to evaluate their independent association with MetS. Consequently, adjusted multivariable models were not performed. Given that several investigated indexes include variables that are also components of the MetS definition, adjustment for these factors may introduce collinearity and overadjustment. Similarly, the superior performance observed for TyG-WC should be interpreted in light of the fact that waist circumference is included both in the index itself and in the IDF definition of MetS, which may have partially inflated its discriminative performance. Future studies should further explore the independent contribution of these indexes using alternative analytical approaches. Finally, the study population consisted exclusively of children and adolescents with obesity who were admitted to a structured three-week in-hospital BWRP. As a consequence, a degree of selection bias cannot be excluded, and the findings may not be fully generalizable to broader pediatric populations with obesity managed in community or outpatient settings.

Nevertheless, this study also presents several strengths. The relatively large sample size enhances the robustness of our findings and enables reliable comparisons of multiple indexes within the same cohort. In addition, the inclusion of both anthropometric and biochemical markers provides a comprehensive evaluation of metabolic dysfunction, while the sex-stratified analysis offers further insight into potential differences in metabolic risk profiles. Furthermore, the simultaneous evaluation of multiple widely used metabolic indexes within a single, well-characterized pediatric cohort represents a key strength, enabling direct comparison of their relative diagnostic performance.

## 5. Conclusions

In conclusion, within a large cohort of children and adolescents with obesity, TyG and TyG-WC demonstrated the highest discriminative ability for identifying metabolic syndrome when directly compared with insulin-based and anthropometric indexes. However, their overall diagnostic performance was moderate, whereas FGIR and QUICKI showed only modest accuracy, and BAI did not provide meaningful discrimination.

These findings suggest the potential clinical value of TyG-based indexes as simple, non-invasive, and readily available tools that can support the early identification of cardiometabolic risk in pediatric obesity, particularly when a pragmatic and scalable screening approach is required. However, the present findings should be interpreted in light of the cross-sectional study design, the absence of external validation of the identified cut-off values, and the lack of comparison with gold-standard measures of insulin resistance.

Further studies are needed to validate these findings in broader, more heterogeneous, community-based, ethnically diverse, and prospectively followed pediatric populations, to establish standardized and generalizable optimal cut-off values, and to assess the integration of these indexes into multimodal risk assessment strategies to improve early identification and targeted intervention in high-risk pediatric subjects. Until such evidence becomes available, these indexes should be considered supportive tools for clinicians in the screening of MetS in high-risk pediatric populations with obesity, without replacing comprehensive clinical evaluation or established diagnostic criteria, such as those proposed by the IDF.

## Figures and Tables

**Table 1:** Main characteristics of the whole study population and of the two subgroups.

	Whole Study Population	MetS+	MetS−	*p* Value
n.	758	211	547	-
Sex (F/M)	454 (59.9%)/304 (40.1%)	105 (49.8%)/106 (50.2%)	349 (63.8%)/198 (36.2%)	<0.0001
Age (yrs)	14.8 ± 2.1	15.4 ± 2.0	14.5 ± 2.1	<0.0001
Tanner stage	3.8 ± 1.4	4.1 ± 1.2	3.7 ± 1.4	<0.0001
WC (cm)	115.2 ± 14.7	122.7 ± 14.3	112.4 ± 13.8	<0.0001
HC (cm)	121.6 ± 12.2	125.2 ± 12.6	120.3 ± 11.8	<0.0001
BW (kg)	101.6 ± 22.7	112.4 ± 23.5	97.4 ± 21.0	<0.0001
Height (cm)	163.0 ± 9.8	166.7 ± 9.6	161.6 ± 9.4	<0.0001
BMI (kg/m^2^)	37.9 ± 6.2	40.2 ± 6.4	37.0 ± 5.9	<0.0001
SBP (mmHg)	125.5 ± 12.6	134.5 ± 11.2	122.0 ± 11.3	<0.0001
DBP (mmHg)	78.5 ± 7.9	82.4 ± 8.2	77.0 ± 7.3	<0.0001
Glucose (mg/dL)	81.4 ± 6.2	81.6 ± 6.8	81.3 ± 6.0	ns
Insulin (mU/L)	14.9 ± 8.7	17.2 ± 8.3	14.0 ± 8.7	<0.0001
T-C (mg/dL)	163.8 ± 31.7	165.0 ± 32.3	163.3 ± 31.3	ns
HDL-C (mg/dL)	42.8 ± 10.5	34.7 ± 5.9	45.9 ± 10.3	<0.0001
TG (mg/dL)	96.6 ± 40.8	125.7 ± 51.3	85.4 ± 29.1	<0.0001
TyG	4.4 ± 0.2	4.6 ± 0.2	4.4 ± 0.2	<0.0001
TyG-WC	512.7 ± 74.3	561.6 ± 70.9	493.7 ± 66.3	<0.0001
BAI	40.6 ± 5.8	40.3 ± 5.9	40.7 ± 5.7	ns
FGIR	8.0 ± 6.7	6.4 ± 5.2	8.6 ± 7.1	<0.0001
QUICKI	0.34 ± 0.03	0.33 ± 0.03	0.34 ± 0.03	<0.0001

Abbreviations: WC, Waist Circumference; HC, Hips Circumference; BW, Body Weight; BMI, Body Mass Index; SBP, Systolic Blood Pressure; DBP, Diastolic Blood Pressure; T-C, Total Cholesterol; HDL-C, HDL Cholesterol; TG, Triglycerides; TyG, Triglyceride Glucose Index; TyG-WC, Triglyceride Glucose Index—Waist Circumference; BAI, Body Adiposity Index; FGIR, Fasting Glucose to Insulin Ratio; QUICKI, Quantitative Insulin Sensitivity Check Index; MetS, Metabolic Syndrome.

**Table 2:** Characteristics of the study population divided by sex. Data partially reported previously in [19].

	Females	Males	*p* Value
n.	454	304	
Age (yrs)	14.8 ± 2.1	14.6 ± 2.2	ns
Tanner stage	4.0 ± 1.3	3.5 ± 1.4	<0.0001
WC (cm)	112.0 ± 13.5	120.1 ± 15.1	<0.0001
HC (cm)	122.3 ± 29.3	120.7 ± 13.2	ns
BW (kg)	97.2 ± 18.7	108.1 ± 26.4	<0.0001
Height (cm)	160.3 ± 7.4	167.0 ± 11.4	<0.0001
BMI (kg/m^2^)	37.7 ± 6.0	38.3 ± 6.4	ns
SBP (mmHg)	123.5 ± 12.1	128.4 ± 12.7	<0.0001
DBP (mmHg)	77.8 ± 7.6	79.7 ± 8.3	<0.01
Glucose (mg/dL)	81.0 ± 6.3	81.9 ± 5.9	ns
Insulin (mU/L)	14.5 ± 8.5	15.4 ± 9.1	ns
T-C (mg/dL)	162.8 ± 31.0	165.3 ± 32.4	ns
HDL-C (mg/dL)	44.3 ± 10.4	40.5 ± 10.3	<0.0001
TG (mg/dL)	93.1 ± 40.1	101.8 ± 41.5	<0.01
MetS (+/−)	105 (23.1%)/349 (76.9%)	106 (34.9%)/198 (65.1%)	<0.0001
TyG	4.4 ± 0.2	4.5 ± 0.2	<0.001
TyG-WC	495.9 ± 68.6	537.8 ± 75.5	<0.0001
BAI	42.3 ± 5.4	38.0 ± 5.4	<0.0001
FGIR	8.1 ± 6.7	7.9 ± 6.8	ns
QUICKI	0.34 ± 0.03	0.33 ± 0.03	ns

Abbreviations: WC, Waist Circumference; HC, Hips Circumference; BW, Body Weight; BMI, Body Mass Index; SBP, Systolic Blood Pressure; DBP, Diastolic Blood Pressure; T-C, Total Cholesterol; HDL-C, HDL Cholesterol; TG, Triglycerides; TyG, Triglyceride Glucose Index; TyG-WC, Triglyceride Glucose Index—Waist Circumference; BAI, Body Adiposity Index; FGIR, Fasting Glucose to Insulin Ratio; QUICKI, Quantitative Insulin Sensitivity Check Index; MetS, Metabolic Syndrome.

**Table 3:** ROC area, cut-off according to Youden Index, Sensitivity, Specificity, Positive Predictive Value, Negative Predictive Value, Positive Likelihood Ratio, and Negative Likelihood Ratio of the five indexes in predicting MetS in the whole study group and in the two subgroups (females and males).

	ROC Area	Cut-Off	Sensitivity	Specificity	PPV	NPV	PLR	NLR
**Whole study population**								
TyG	0.75 (0.71–0.79)	4.54	60.2% (53.2–66.8%)	80.2% (77.2–84.0%)	54.7% (49.4–61.7%)	84.0% (79.9–86.8%)	3.14 (2.56–3.85)	0.50 (0.42–0.58)
TyG-WC	0.76 (0.73–0.80)	530.07	64.9% (58.1–71.4%)	75.1% (71.3–78.7%)	50.2% (45.3–57.5%)	84.7% (80.6–87.2%)	2.61 (2.19–3.12)	0.47 (0.39–0.56)
BAI	0.48 (0.43–0.53)	45.39	19.4% (14.3–25.4%)	82.4% (79.0–85.5%)	29.9% (25.5–37.6%)	72.6% (64.8–77.0%)	1.11 (0.80–1.54)	0.98 (0.91–1.01)
FGIR	0.64 (0.59–0.68)	6.2	71.1% (64.5–77.1%)	54.3% (50.0–58.5%)	37.5% (33.6–45.1%)	83.0% (78.2–85.3%)	1.55 (1.37–1.76)	0.53 (0.43–0.67)
QUICKI	0.63 (0.58–0.67)	0.34	75.4% (69.0–81.0%)	49.7% (45.5–54.0%)	36.6% (32.8–44.7%)	84.0% (79.2–86.1%)	1.50 (1.34–1.68)	0.50 (0.39–0.64)
**Females**								
TyG	0.76 (0.70–0.82)	4.54	61.0% (50.9–70.3%)	84.0% (79.7–87.6%)	53.3% (46.1–63.4%)	87.7% (82.6–90.6%)	3.80 (2.86–5.05)	0.47 (0.37–0.59)
TyG-WC	0.77 (0.72–0.82)	500.40	76.2% (66.9–84.0%)	67.3% (62.1–72.2%)	41.2% (35.8–53.4%)	90.4% (85.6–92.2%)	2.33 (1.94–2.81)	0.35 (0.25–0.50)
BAI	0.55 (0.50–0.62)	40.78	63.8% (53.9–73.0%)	47.3% (41.9–52.7%)	26.7% (22.7–35.8%)	81.3% (74.2–84.3%)	1.21 (1.02–1.44)	0.77 (0.58–1.01)
FGIR	0.64 (0.58–0.70)	6.27	70.5% (60.8–79.0%)	55.9% (50.5–61.2%)	32.5% (27.9–43.1%)	86.3% (80.3–88.7%)	1.60 (1.35–1.90)	0.53 (0.39–0.72)
QUICKI	0.63 (0.57–0.69)	0.34	75.2% (65.9–83.1%)	50.4% (45.1–55.8%)	31.3% (26.9–42.6%)	87.1% (81.1–89.4%)	1.52 (1.30–1.77)	0.49 (0.35–0.70)
**Males**								
TyG	0.72 (0.66–0.78)	4.54	61.3% (51.4–70.6%)	73.7% (67.0–79.7%)	55.6% (47.5–65.5%)	78.1% (70.3–83.3%)	2.34 (1.77–3.08)	0.53 (0.41–0.68)
TyG-WC	0.72 (0.66–0.78)	529.39	73.6% (64.1–81.7%)	62.1% (55.0–68.9%)	51.0% (43.6–62.5%)	81.5% (73.8–85.6%)	1.94 (1.57–2.40)	0.43 (0.30–0.60)
BAI	0.56 (0.50–0.62)	32.96	86.8% (78.8–92.6%)	16.7% (11.8–22.6%)	35.8% (27.1–51.5%)	70.2% (57.2–77.5%)	1.04 (0.95–1.15)	0.79 (0.44–1.41)
FGIR	0.63 (0.57–0.70)	5.95	69.8% (60.1–78.3%)	55.6% (48.3–62.6%)	45.7% (38.6–56.8%)	77.5% (69.2–82.2%)	1.57 (1.29–1.92)	0.54 (0.40–0.75)
QUICKI	0.62 (0.56–0.69)	0.33	73.6% (64.1–81.7%)	52.0% (44.8–59.2%)	45.1% (38.1–56.8%)	78.6% (70.2–83.1%)	1.53 (1.28–1.84)	0.51 (0.30–0.72)

Abbreviations: TyG, Triglyceride Glucose Index; TyG-WC, Triglyceride Glucose Index—Waist Circumference; BAI, Body Adiposity Index; FGIR, Fasting Glucose to Insulin Ratio; QUICKI, Quantitative Insulin Sensitivity Check Index; MetS, Metabolic Syndrome; PPV, Positive Predictive Value; NPV, Negative Predictive Value; PLR, Positive Likelihood Ratio; NLR, Negative Likelihood Ratio.

## Data Availability

The datasets generated and analyzed for this study will be uploaded to www.zenodo.org and will be available from the corresponding author upon reasonable request.

## Supplementary Materials

The following supporting information can be downloaded at: https://www.mdpi.com/article/10.3390/metabo16060415/s1, Supplementary Figure S1: Participant flow chart; Supplementary Table S1: Correlation between each index and anthropometric and clinical characteristics in the whole study population and in the two subgroups (females and males).
