# Supplementary material for: Discriminative Ability of TyG, TyG-WC, BAI, FGIR, and QUICKI Indexes in Identifying Metabolic Syndrome in a Pediatric Population with Obesity
Source: Metabolites. 2026 Jun 14;16(6):415. doi: 10.3390/metabo16060415 (PMC13303531; doi:10.3390/metabo16060415)
Supplement: Supplementary file 1 [file metabolites-16-00415-s001.zip › metabolites-4332402-supplementary.pdf]

Supplementary Material

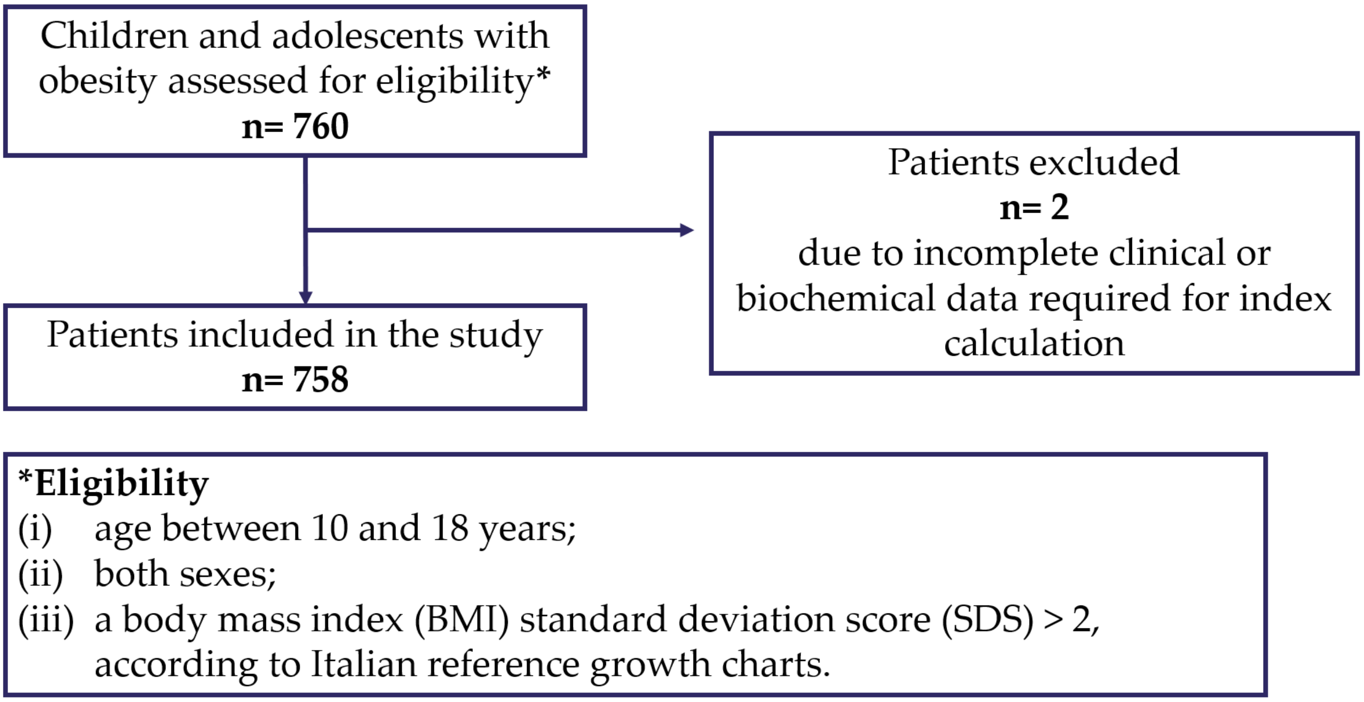

Supplementary Figure S1. Participant flow chart.

Supplementary Table S1. Correlation between each index and anthropometric and clinical characteristics in the whole study population and in the two subgroups (females and males).

		Age	Height	BW	BMI	WC	HC	SBP	DBP	MetS
**Whole study population**										
TYG	r	0.06	0.11	0.19	0.18	0.21	0.08	0.17	0.12	0.41
	p-value	ns	<0.01	<0.001	<0.001	<0.001	<0.05	<0.001	<0.01	<0.001
TYG-WC	r	0.33	0.46	0.80	0.74	0.95	0.67	0.39	0.34	0.41
	p-value	<0.001	<0.001	<0.001	<0.001	<0.001	<0.001	<0.001	<0.001	<0.001
BAI	r	0.01	0.44	0.25	0.66	0.31	0.60	0.08	0.16	0.03
	p-value	ns	<0.001	<0.001	<0.001	<0.001	<0.001	<0.05	<0.001	ns
FGIR	r	0.03	0.12	0.26	0.27	0.26	0.18	0.17	0.16	0.15
	p-value	ns	<0.01	<0.001	<0.001	<0.001	<0.001	<0.001	<0.001	<0.001
QUICKI	r	0.01	0.14	0.32	0.34	0.32	0.22	0.21	0.19	0.18
	p-value	ns	<0.001	<0.001	<0.001	<0.001	<0.001	<0.001	<0.001	<0.001
**Females**										
TYG	r	0.0	0.01	0.18	0.21	0.21	0.10	0.15	0.09	0.42
	p-value	ns	ns	<0.001	<0.001	<0.001	<0.05	<0.01	ns	<0.001
TYG-WC	r	0.21	0.24	0.72	0.72	0.95	0.66	0.31	0.32	0.43
	p-value	<0.001	<0.001	<0.001	<0.001	<0.001	<0.001	<0.001	<0.001	<0.001
BAI	r	0.07	0.33	0.52	0.80	0.54	0.72	0.21	0.24	0.09
	p-value	ns	<0.001	<0.001	<0.001	<0.001	<0.001	<0.001	<0.001	ns
FGIR	r	0.07	0.03	0.19	0.24	0.20	0.12	0.13	0.13	0.14
	p-value	ns	ns	<0.001	<0.001	<0.001	<0.05	<0.01	<0.01	<0.001
QUICKI	r	0.11	0.02	0.25	0.31	0.27	0.17	0.17	0.16	0.17
	p-value	<0.05	ns	<0.001	<0.001	<0.001	<0.001	<0.001	<0.05	<0.001
**Males**										
TYG	r	0.17	0.13	0.16	0.12	0.16	0.08	0.15	0.13	0.37
	p-value	<0.01	<0.05	<0.01	<0.05	<0.01	ns	<0.05	<0.05	<0.001
TYG-WC	r	0.56	0.56	0.86	0.81	0.95	0.79	0.43	0.33	0.35
	p-value	<0.001	<0.001	<0.001	<0.001	<0.001	<0.001	<0.001	<0.001	<0.001
BAI	r	0.11	0.40	0.22	0.62	0.34	0.51	0.09	0.17	0.07
	p-value	<0.05	<0.001	<0.001	<0.001	<0.001	<0.001	ns	<0.001	ns
FGIR	r	0.18	0.27	0.34	0.31	0.34	0.28	0.22	0.20	0.16
	p-value	<0.001	<0.001	<0.001	<0.001	<0.001	<0.001	<0.001	<0.001	<0.01
QUICKI	r	0.17	0.28	0.39	0.37	0.39	0.31	0.24	0.24	0.18
	p-value	<0.01	<0.001	<0.001	<0.001	<0.001	<0.001	<0.001	<0.001	<0.001

Abbreviations: WC, Waist Circumference; HC, Hips Circumference; BW, Body Weight; BMI, Body Mass Index; SBP, Systolic Blood Pressure; DBP, Diastolic Blood Pressure TyG, Triglyceride Glucose Index; TyG-WC, Triglyceride Glucose Index-Waist Circumference ratio; BAI, Body Adiposity Index; FGIR, Fasting Glucose to Insulin Ratio; QUICKI, Quantitative Insulin Sensitivity Check Index; MetS, Metabolic Syndrome.
